# Associations of Emotional Intelligence and Ethical Climate With Job Involvement Among Operating Room Personnel: A Cross‐Sectional Study

**DOI:** 10.1155/jonm/4920152

**Published:** 2026-10-05

**Authors:** Fatemeh Vashahi Torfi, Armin Fereidouni, Amirali Alizadeh, Samira Mahmoudi

**Affiliations:** ^1^ Student Research Committee Shiraz University of Medical Sciences Shiraz Iran sums.ac.ir; ^2^ Department of Operating Room Technology School of Nursing and Midwifery Shiraz University of Medical Sciences Shiraz Iran deakin.edu.au; ^3^ Research Center for Evidence-Based Health Management Maragheh University of Medical Sciences Maragheh Iran mrgums.ac.ir; ^4^ Department of Anesthesiology School of Nursing and Midwifery Shiraz University of Medical Sciences Shiraz Iran sums.ac.ir

**Keywords:** emotional intelligence, ethical climate, job involvement, operating room personnel, perioperative practice

## Abstract

**Background:**

Operating room practice requires personnel to remain actively committed to their professional roles while managing complex clinical tasks, close interdisciplinary coordination, and substantial responsibility for patient safety. Despite the importance of such work‐related involvement, its association with personal emotional capabilities and the ethical characteristics of the workplace has received limited attention among operating room personnel. This study evaluated job involvement and examined its relationships with emotional intelligence and perceived ethical climate.

**Methods:**

This cross‐sectional study was conducted in 2023 among operating room personnel employed in five teaching hospitals affiliated with Shiraz University of Medical Sciences. The hospitals constituted sampling clusters, and eligible personnel within each hospital were recruited through convenience sampling. A total of 348 participants completed the Schutte Emotional Intelligence Scale, Olson’s Hospital Ethical Climate Survey, and Kanungo’s Job Involvement Questionnaire. Pearson correlations and multiple linear regression were used, and hospital‐level clustering was examined using random‐intercept mixed‐model sensitivity analyses.

**Results:**

Of 370 initially recruited personnel, 348 were included in the complete‐case analysis (94.1%). Mean scores were 106.32 ± 20.85 for emotional intelligence, 82.50 ± 22.00 for ethical climate, and 29.77 ± 9.21 for job involvement. Job involvement was slightly below the theoretical midpoint of 30 despite a generally favorable perceived ethical climate. Job involvement correlated positively with ethical climate (*r* = 0.30, *p* < 0.001) and emotional intelligence (*r* = 0.22, *p* < 0.001); emotional intelligence and ethical climate were not significantly correlated (*r* = 0.088, *p* = 0.102). In the fully adjusted model, ethical climate (standardized *β* = 0.277, *p* < 0.001) and emotional intelligence (standardized *β* = 0.190, *p* < 0.001) remained independently associated with job involvement. The model explained 15.8% of the variance (*R* ^2^ = 0.158), and results were materially unchanged after accounting for hospital‐level clustering.

**Conclusion:**

Job involvement among operating room personnel was independently associated with perceived ethical climate and emotional intelligence, with ethical climate showing the stronger association. These individual and organizational resources may represent distinct correlates of psychological involvement in work. Given the cross‐sectional design, causal inference is not warranted.

**Implications for Nursing Management:**

Nursing managers may consider strategies to strengthen ethical workplace climates and emotional competencies among operating room personnel through supportive leadership, transparent and equitable decision‐making, structured opportunities to address ethical concerns, targeted training, and effective communication. Continuing professional development focused on self‐awareness, emotional regulation, empathy, and interpersonal communication may complement organizational initiatives that promote fairness, mutual respect, psychological support, and accessible mechanisms for discussing ethical concerns. A dual‐level approach addressing personal and organizational resources warrants prospective evaluation; the present cross‐sectional findings do not establish the effectiveness of any specific intervention.

## 1. Introduction

Operating rooms are complex, high‐acuity clinical settings in which the actions of healthcare personnel influence patient safety, procedural efficiency, and clinical outcomes [1]. Operating room personnel prepare the surgical environment, organize equipment and instruments, maintain aseptic conditions, support infection‐prevention measures, coordinate with members of the surgical team, and provide perioperative care [2]. Effective performance therefore depends not only on technical competence but also on personnel’s capacity to remain psychologically invested in their professional roles while functioning within demanding interpersonal and organizational conditions.

Operating room practice also involves substantial occupational demands. Personnel frequently work under time pressure, manage serious clinical responsibilities, encounter unpredictable or critical situations, perform irregular shifts, and make rapid decisions while coordinating with multiple professional groups. Within Job Demands–Resources (JD‐R) theory, these characteristics can be conceptualized as job demands that require sustained physical, emotional, and cognitive effort; their impact depends partly on the personal and organizational resources available to employees [3, 4].

One work‐related construct that may be particularly relevant in this context is job involvement. Job involvement reflects the degree to which individuals psychologically identify with their job and consider it an important component of their self‐concept [5]. Although related to broader concepts such as work engagement, job involvement specifically concerns psychological identification with one’s job and should be treated as a distinct work‐related construct. In nursing, job involvement has been linked to organizational support, professional attitudes, and caring‐related outcomes [6–8].

Beyond the demands inherent in operating room practice, employees’ investment in work may be influenced by personal resources and the organizational environment. Emotional intelligence is one such personal resource and involves recognizing emotional cues, understanding their meaning, regulating one’s own emotional responses, and responding appropriately to others [9]. In healthcare settings, emotional intelligence has been associated with coping, work engagement, performance, and safe professional practice [10–13]. From a JD‐R perspective, emotional intelligence may help personnel manage emotional and interpersonal demands and thereby support psychological identification with professional roles.

Organizational conditions may be equally important. Ethical climate refers to employees’ shared perceptions of the ethical standards, expectations, and practices that shape conduct and decision‐making within an organization [14]. Favorable ethical climates have been associated with more positive occupational attitudes and lower burnout among healthcare workers [8, 15, 16]. Within the JD‐R theory, ethical climate may be conceptualized as an organizational resource that provides clearer ethical expectations, supportive professional relationships, and a more predictable context for addressing workplace concerns.

Importantly, emotional intelligence and ethical climate represent conceptually distinct levels of resources. Emotional intelligence primarily reflects an individual‐level capacity, whereas ethical climate reflects perceptions of the organizational environment. Their contributions to job involvement may therefore be complementary rather than redundant, and a strong correlation between the two constructs is not necessarily expected. This distinction provides a theoretical basis for examining whether each is independently associated with job involvement.

Most available evidence on job involvement and related work attitudes has been generated in general nursing populations, while evidence simultaneously examining an individual emotional resource and an organizational ethical resource among operating room personnel remains limited. Accordingly, this study aimed to determine the level of job involvement among operating room personnel and examine its associations with emotional intelligence and ethical climate within a JD‐R‐informed conceptual perspective.

**Hypothesis 1.**

*(H1):* Emotional intelligence is positively associated with job involvement among operating room personnel.

**Hypothesis 2.**

*(H2):* Ethical climate is positively associated with job involvement among operating room personnel.

**Hypothesis 3.**

*(H3):* Emotional intelligence and ethical climate are each independently and positively associated with job involvement when considered simultaneously and after adjustment for demographic and occupational characteristics.

## 2. Materials and Methods

### 2.1. Study Design and Setting

A descriptive cross‐sectional study was conducted in 2023 among operating room personnel employed in five teaching hospitals affiliated with Shiraz University of Medical Sciences, Shiraz, Iran. The participating hospitals were Namazi, Khalili, Shahid Rajaei, Shahid Chamran, and Shahid Faghihi hospitals.

### 2.2. Participants and Sampling

Participants were recruited using a two‐stage sampling approach. In the first stage, the five participating teaching hospitals constituted the sampling clusters. In the second stage, eligible operating room personnel within each hospital were recruited using convenience sampling; participant selection at the individual level was therefore nonprobabilistic.

The original recruitment target was determined using the formula for estimating a population mean and the standard deviation reported by Pérez‐Fuentes et al. [17]. With a 95% confidence level and the prespecified precision, the minimum required sample size was 307 participants. To account for potential nonresponse and incomplete questionnaires, this number was increased by approximately 20%, resulting in a target sample of 370 personnel.

Because multiple linear regression constituted an important component of the analysis, an additional sample‐adequacy assessment was performed using G∗Power for a fixed‐model multiple linear regression. Assuming a small effect size (*f* ^2^ = 0.05), *α* = 0.05, statistical power of 0.80, and eight predictors, the minimum required sample size was 313 participants. The final analytic sample of 348 participants exceeded this requirement.

### 2.3. Eligibility Criteria

Personnel were eligible if they (1) held at least an associate degree in nursing or operating room technology; (2) had at least 1 year of professional experience in an operating room; (3) self‐reported no history of a diagnosed psychiatric disorder; and (4) voluntarily agreed to participate and provided written informed consent.

During data screening, 22 participants were excluded from the final analysis. Fourteen questionnaires were excluded because more than 10% of item‐level responses were missing, and eight participants were excluded because they were transferred to another ward or hospital during the data‐collection period. No missing‐data imputation was performed; final analyses used complete cases (*N* = 348).

### 2.4. Recruitment and Data Collection Procedure

After administrative authorization was obtained from the Vice Chancellor for Research of Shiraz University of Medical Sciences, the researcher coordinated with operating room managers at each participating hospital. Following approval from hospital authorities, staff lists and work schedules were reviewed throughout the data‐collection period to identify potentially eligible personnel during scheduled shifts. Potential participants were approached in person during their scheduled work shifts. The researcher explained the study objectives, procedures, voluntary nature of participation, and confidentiality of the collected information. Those who agreed provided written informed consent and received the questionnaires, which were returned within 24 h.

### 2.5. Data Collection Instruments

Data collection involved a researcher‐prepared demographic form and three standardized questionnaires measuring emotional intelligence, perceived ethical climate, and job involvement.

#### 2.5.1. Demographic and Occupational Characteristics

The demographic form obtained age, sex, marital status, educational attainment, professional field, and length of professional experience in the operating room.

#### 2.5.2. Schutte Emotional Intelligence Scale

Emotional intelligence was assessed using the Schutte Emotional Intelligence Scale [9]. The Persian version used in this study contains 33 items covering self‐awareness, self‐regulation, self‐motivation, empathy, and social skills. Responses are recorded on a five‐point Likert scale. Items were scored according to the validated Persian version, including reverse coding where required. Total scores range from 33 to 165, with higher scores indicating greater emotional intelligence. Previous Persian‐language evaluations have reported acceptable reliability [18, 19]. In the present sample, Cronbach’s alpha was 0.88 (95% CI [0.85, 0.90]); alpha coefficients for self‐awareness, self‐regulation, self‐motivation, empathy, and social skills were 0.81, 0.76, 0.79, 0.82, and 0.75, respectively.

#### 2.5.3. Hospital Ethical Climate Survey

Perceived ethical climate was measured using Olson’s Hospital Ethical Climate Survey, a 26‐item instrument covering ethical relationships with colleagues, physicians, managers, patients, and the hospital [14]. Items are rated on a five‐point scale and scored according to the validated version, including reverse coding where applicable. Total scores range from 26 to 130, with higher scores indicating a more favorable perceived ethical climate. Scores below 78 have been used to indicate an unfavorable climate, whereas scores of 78 or higher indicate a favorable climate. Iranian studies have supported the instrument’s reliability [20, 21]. Cronbach’s alpha in the current sample was 0.91 (95% CI [0.88, 0.93]).

#### 2.5.4. Kanungo’s Job Involvement Questionnaire

Job involvement was evaluated using Kanungo’s 10‐item Job Involvement Questionnaire [5]. The instrument uses a five‐point Likert response format and yields total scores from 10 to 50, with higher scores reflecting stronger psychological identification with one’s job. In the current sample, Cronbach’s alpha was 0.85 (95% CI [0.82, 0.87]).

### 2.6. Statistical Analysis

Data were analyzed using IBM SPSS Statistics for Windows, Version 22.0 (IBM Corp., Armonk, NY, USA). Continuous variables are reported as means and standard deviations and categorical variables as frequencies and percentages. Medians, interquartile ranges, and observed ranges were additionally calculated for the three principal study variables. Analyses were conducted on complete cases (*N* = 348), and no missing‐data imputation was performed.

Pearson correlation coefficients were calculated to examine bivariate relationships among emotional intelligence, ethical climate, and job involvement. Linearity and potentially influential observations were assessed graphically, and 95% confidence intervals were calculated for correlation coefficients.

Because all three principal constructs were assessed using self‐report questionnaires at the same time point, potential common method bias was evaluated using Harman’s single‐factor test. All 69 questionnaire items were entered simultaneously into an unrotated factor analysis, and the percentage of total variance accounted for by the first factor was examined. A first‐factor variance below 50% was interpreted as indicating no dominant single‐factor structure, while recognizing the limitations of this diagnostic [22].

Multiple linear regression was used to examine the independent associations of emotional intelligence and ethical climate with job involvement. In Model 1, emotional intelligence and ethical climate were entered simultaneously as the focal predictors. Model 2 additionally included age, work experience, sex, marital status, educational level, and professional field. A complementary hierarchical analysis entered demographic and occupational covariates in the first block and emotional intelligence and ethical climate in the second block to quantify incremental explained variance. Categorical variables were dummy coded using female, single, bachelor’s degree, and operating room technology as reference categories; age and work experience were entered as continuous variables.

Regression findings are reported using unstandardized coefficients (*B*), standard errors, standardized coefficients (*β*), *t* statistics, *p* values, and 95% confidence intervals. Overall model performance was evaluated using *R* ^2^, adjusted *R* ^2^, and the overall *F* statistic. For the hierarchical analysis, Δ*R* ^2^ and the *F*‐change statistic were examined. Regression assumptions were evaluated using residual‐versus‐predicted plots, probability plots of residuals, Cook’s distance, variance inflation factors, and tolerance statistics.

Because participants were recruited from five hospitals, hospital‐level clustering was evaluated using an empty random‐intercept model to estimate the intraclass correlation coefficient (ICC). Random‐intercept linear mixed models with hospital as the clustering variable were then fitted as sensitivity analyses to determine whether accounting for hospital‐level clustering materially altered the estimated associations of emotional intelligence and ethical climate with job involvement.

Exploratory subgroup analyses examined differences in job involvement by sex, marital status, educational level, and professional field using independent‐samples *t* tests. Differences across the five hospitals were assessed using one‐way analysis of variance. All statistical tests were two‐sided, with *p* < 0.05 considered statistically significant.

### 2.7. Ethical Considerations

Ethical approval was obtained from the Ethics Committee of Shiraz University of Medical Sciences (IR.SUMS.NUMIMG.REC.1402.047). All participants received study information and provided written informed consent. Participation was voluntary, withdrawal was permitted without consequence, no personally identifiable information was recorded, and data were anonymized before analysis.

## 3. Results

Of 370 operating room personnel initially recruited, 22 were excluded from the final analysis: 14 because more than 10% of item‐level responses were missing and eight because they were transferred to another ward or hospital during data collection. The final analytic sample comprised 348 participants (94.1% of the initially recruited sample). Mean age was 30.24 ± 4.54 years. Most participants were female (78.4%), single (58.6%), and graduates of operating room technology programs (73.0%) (Table 1).

**TABLE 1:** Demographic and occupational characteristics of operating room personnel (*N* = 348).

Variable	Category	*N*/mean ± SD	%
Sex	Male	75	21.6
	Female	273	78.4

Marital status	Single	204	58.6
	Married	144	41.4

Education level	Bachelor’s degree	316	90.8
	Master’s degree	32	9.2

Professional field	Nursing	94	27.0
	Operating room technology	254	73.0

Age (years)	—	30.24 ± 4.54	—

Work experience (years)	—	6.14 ± 4.36	—

Mean emotional intelligence was 106.32 ± 20.85. Self‐awareness had the highest subscale mean (27.99 ± 6.46), whereas social skills had the lowest (13.44 ± 4.82). Mean ethical climate was 82.50 ± 22.00, above the predefined cutoff of 78 and therefore consistent with a generally favorable perceived ethical climate. Mean job involvement was 29.77 ± 9.21, slightly below the theoretical midpoint of 30 (Table 2).

**TABLE 2:** Descriptive statistics for emotional intelligence, ethical climate, and job involvement.

Variable	Mean ± SD	Median (IQR)	Observed range	Theoretical range
Emotional intelligence (total)	106.32 ± 20.85	105 (28)	45–158	33–165
Self‐awareness	27.99 ± 6.46	—	—	—
Self‐regulation	23.89 ± 5.55	—	—	—
Self‐motivation	23.04 ± 6.31	—	—	—
Empathy	17.94 ± 5.88	—	—	—
Social skills	13.44 ± 4.82	—	—	—
Ethical climate	82.50 ± 22.00	83 (30)	35–128	26–130
Job involvement	29.77 ± 9.21	29 (12)	12–48	10–50

The three instruments demonstrated good internal consistency in the present sample (Cronbach’s *α* = 0.88 for emotional intelligence, 0.91 for ethical climate, and 0.85 for job involvement). Harman’s single‐factor analysis showed adequate factorability (KMO = 0.864; Bartlett’s *χ* ^2^ (2346) = 11,452.38, *p* < 0.001). The first unrotated factor accounted for 31.42% of total variance, below the 50% criterion, indicating no dominant single‐factor structure.

Job involvement correlated positively with ethical climate (*r* = 0.30, *p* < 0.001, 95% CI [0.20, 0.39]) and emotional intelligence (*r* = 0.22, *p* < 0.001, 95% CI [0.12, 0.32]). Emotional intelligence was not significantly correlated with ethical climate (*r* = 0.088, *p* = 0.102, 95% CI [−0.02, 0.19]) (Table 3). Figures 1 and 2 display the corresponding bivariate relationships.

**TABLE 3:** Pearson correlations among ethical climate, job involvement, and emotional intelligence.

Variable	Ethical climate, *r* (95% CI)	*p*	Job involvement, *r* (95% CI)	*p*
Job involvement	0.30 [0.20, 0.39]	< 0.001	—	—
Emotional intelligence	0.088 [−0.02, 0.19]	0.102	0.22 [0.12, 0.32]	< 0.001

**FIGURE 1:**
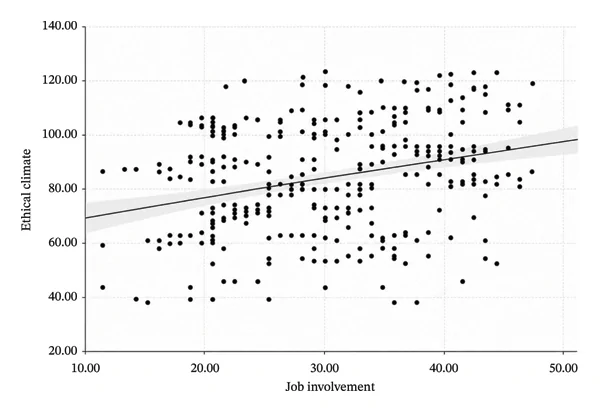
Scatterplot showing the relationship between job involvement and perceived ethical climate, with the fitted linear regression line and 95% confidence interval.

**FIGURE 2:**
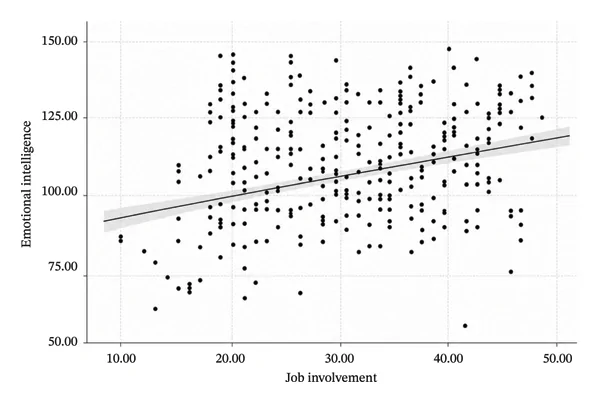
Scatterplot showing the relationship between job involvement and emotional intelligence, with the fitted linear regression line and 95% confidence interval.

When emotional intelligence and ethical climate were entered simultaneously in Model 1, both were independently and positively associated with job involvement. Ethical climate showed the stronger association (*B* = 0.118, SE = 0.021, standardized *β* = 0.283, 95% CI [0.077, 0.159], *p* < 0.001), while emotional intelligence was also positively associated (*B* = 0.086, SE = 0.022, standardized *β* = 0.195, 95% CI [0.043, 0.129], *p* < 0.001). Model 1 explained 14.5% of the variance in job involvement (*R* ^2^ = 0.145, adjusted *R* ^2^ = 0.140; *F* (2, 345) = 29.25, *p* < 0.001).

In the fully adjusted model controlling for age, work experience, sex, marital status, educational level, and professional field, both associations remained statistically significant. Ethical climate remained the stronger independent correlate (*B* = 0.116, SE = 0.021, standardized *β* = 0.277, 95% CI [0.075, 0.157], *p* < 0.001), followed by emotional intelligence (*B* = 0.084, SE = 0.023, standardized *β* = 0.190, 95% CI [0.039, 0.129], *p* < 0.001). None of the demographic or occupational covariates was significantly associated with job involvement. The fully adjusted model explained 15.8% of the variance (*R* ^2^ = 0.158, adjusted *R* ^2^ = 0.138; *F* (8, 339) = 7.95, *p* < 0.001) (Tables 4 and 5). In the complementary hierarchical analysis, adding emotional intelligence and ethical climate after demographic and occupational covariates increased explained variance by 14.0% (Δ*R* ^2^ = 0.140; *F*‐change (2, 339) = 28.18, *p* < 0.001).

**TABLE 4:** Multiple linear regression analyses of factors associated with job involvement: Model 1: Emotional intelligence and ethical climate entered simultaneously.

Predictor	*B*	SE	*β*	*t*	95% CI for *B*	*p*
Emotional intelligence	0.086	0.022	0.195	3.910	[0.043, 0.129]	< 0.001
Ethical climate	0.118	0.021	0.283	5.619	[0.077, 0.159]	< 0.001

*Note:* Model fit: *R* ^2^ = 0.145; adjusted *R* ^2^ = 0.140; *F* (2, 345) = 29.25; *p* < 0.001.

**TABLE 5:** Multiple linear regression analyses of factors associated with job involvement: Model 2: Fully adjusted model.

Predictor	*B*	SE	*β*	*t*	95% CI for *B*	*p*
Emotional intelligence	0.084	0.023	0.190	3.652	[0.039, 0.129]	< 0.001
Ethical climate	0.116	0.021	0.277	5.524	[0.075, 0.157]	< 0.001
Age (years)	0.045	0.130	0.022	0.346	[−0.211, 0.301]	0.730
Work experience (years)	0.072	0.135	0.034	0.533	[−0.194, 0.338]	0.594
Sex (male)	0.845	1.150	0.038	0.735	[−1.417, 3.107]	0.463
Marital status (married)	−0.512	1.015	−0.027	−0.504	[−2.508, 1.484]	0.614
Education (master’s degree)	0.315	1.680	0.010	0.187	[−2.990, 3.620]	0.851
Professional field (nursing)	−0.150	1.100	−0.007	−0.136	[−2.314, 2.014]	0.892

*Note:* Model fit: *R* ^2^ = 0.158; adjusted *R* ^2^ = 0.138; *F* (8, 339) = 7.95; *p* < 0.001. Reference categories were female, single, bachelor’s degree, and operating room technology. In the complementary hierarchical analysis, Δ*R* ^2^ = 0.140; *F*‐change (2, 339) = 28.18; *p* < 0.001.

Regression diagnostics did not indicate material violations of model assumptions. Residual probability and residual‐versus‐predicted plots supported approximate normality and homoscedasticity. VIFs ranged from 1.04 to 3.28, with tolerance values ≥ 0.304. Although age and work experience were strongly correlated (*r* = 0.831, *p* < 0.001), their VIF values were 3.25 and 3.28. Maximum Cook’s distance was 0.04, indicating no highly influential observation.

Hospital‐level clustering was small (ICC = 0.024), indicating that 2.4% of the variance in job involvement was attributable to between‐hospital differences. Adjusted random‐intercept mixed‐model estimates were materially consistent with OLS estimates: ethical climate remained positively associated with job involvement (*B* = 0.115, SE = 0.022, 95% CI [0.072, 0.158], *p* < 0.001), as did emotional intelligence (*B* = 0.083, SE = 0.023, 95% CI [0.038, 0.128], *p* < 0.001).

Exploratory subgroup analyses showed no significant differences in job involvement by sex (*p* = 0.302), marital status (*p* = 0.425), educational level (*p* = 0.654), or professional field (*p* = 0.664). Job involvement also did not differ significantly across the five hospitals (*F* (4, 343) = 1.124, *p* = 0.345).

## 4. Discussion

This study examined job involvement among operating room personnel and evaluated its associations with emotional intelligence and perceived ethical climate. Participants reported a generally favorable ethical climate, while mean job involvement was slightly below the theoretical midpoint. Both emotional intelligence and ethical climate were independently and positively associated with job involvement after adjustment for demographic and occupational characteristics, with ethical climate showing the stronger association. These findings are consistent with a JD‐R perspective in which emotional intelligence may function as a personal resource and ethical climate as an organizational resource. No significant relationship was observed between emotional intelligence and ethical climate, suggesting that these resources may operate through relatively distinct individual‐ and organizational‐level pathways.

The emotional intelligence score observed in this study is broadly consistent with recent evidence among critical care and operating room nurses [10, 23]. Operating room personnel routinely encounter emotionally demanding situations that require composure, regulation of emotional responses, effective communication, and timely decision‐making in multidisciplinary teams. Within the JD‐R theory, emotional intelligence may therefore represent a personal resource that assists personnel in managing emotional and interpersonal demands. However, the cross‐sectional design cannot establish whether occupational exposure develops such competencies or whether personnel with stronger emotional competencies adapt more effectively to the operating room environment.

Participants perceived the ethical climate of their workplaces as generally favorable. Recent nursing studies continue to demonstrate that ethical climate is meaningfully related to work attitudes and organizational outcomes, including job involvement and work engagement [8, 16]. Differences across settings may reflect organizational culture, leadership, workload, staffing, participant characteristics, or measurement approaches. From a JD‐R perspective, ethical climate may provide an organizational resource through clearer ethical expectations, supportive professional relationships, and more predictable pathways for addressing ethically challenging situations.

Despite a favorable ethical climate, mean job involvement was 29.77, only 0.23 points below the theoretical midpoint of 30. This should not be interpreted as markedly low job involvement; rather, relatively favorable personal and organizational resources did not correspond to substantially elevated psychological involvement in work. Nursing work environments include multiple demands and resources, and evidence indicates that workload, staffing, leadership, professional autonomy, and practice environment characteristics are related to burnout and engagement [24, 25]. In operating room settings specifically, high workload, hierarchical relationships, limited autonomy, and insufficient organizational support have been described as salient contributors to burnout and professional detachment. These unmeasured factors may have attenuated the benefits of emotional intelligence and ethical climate, but they should be regarded as plausible hypotheses rather than demonstrated mechanisms in the present study.

The positive association between ethical climate and job involvement indicates that personnel who perceive their organizational environment as more ethically supportive also tend to report greater psychological involvement in work. This aligns with recent evidence directly linking ethical climate to job involvement among nurses [8] and with evidence linking ethical climate to work engagement [16]. Although work engagement and job involvement are conceptually related but distinct constructs, these findings collectively support the relevance of the ethical work environment to employees’ work‐related attitudes. From a JD‐R perspective, fairness, respectful professional relationships, organizational support, and transparent decision‐making may constitute organizational resources that help explain this association. The cross‐sectional design, however, precludes inference about directionality.

Emotional intelligence was also positively associated with job involvement. Previous studies have predominantly examined work engagement rather than Kanungo’s specific construct of job involvement, but they similarly report positive relationships between emotional intelligence and favorable work‐related outcomes among nurses [13, 17]. Within JD‐R theory, emotional intelligence may function as a personal resource that helps personnel regulate emotional reactions, resolve interpersonal challenges, and maintain constructive relationships within surgical teams. These mechanisms remain theoretical explanations rather than established causal pathways in this cross‐sectional study.

No significant association was found between emotional intelligence and ethical climate. Emotional intelligence is primarily an individual attribute involving emotional perception, understanding, and regulation, whereas ethical climate reflects perceptions of organizational norms, practices, and decision‐making. Ethical‐climate perceptions may be shaped more directly by leadership, organizational justice, workplace culture, managerial behavior, and interprofessional relationships. The weak correlation between emotional intelligence and ethical climate therefore supports the view that personal and organizational resources may contribute to job involvement through relatively distinct pathways. This limited overlap also helps explain why both variables retained independent associations with job involvement when entered simultaneously. Recent evidence linking ethical climate, organizational justice, and work engagement further supports the relevance of these distinct organizational pathways [26].

### 4.1. Practical Implications for Nursing Management

Given the positive association between ethical climate and job involvement, managers may consider organizational strategies aimed at strengthening fairness, mutual respect, trust, supportive leadership, and transparency. Practical approaches may include accessible mechanisms for raising ethical concerns, structured multidisciplinary discussions of ethically challenging situations, and clearly communicated escalation pathways. Such approaches should be prospectively evaluated rather than assumed to increase job involvement.

Because emotional intelligence was independently associated with job involvement, managers may also consider structured continuing professional development addressing self‐awareness, self‐regulation, empathy, communication, and interpersonal skills, accompanied by appropriate pre‐ and post‐intervention evaluation. A dual‐level approach that addresses both personal and organizational resources is consistent with JD‐R theory and may be more useful than focusing exclusively on either domain. The present observational findings, however, do not establish the effectiveness of any specific intervention.

### 4.2. Strengths and Limitations

This study focused on operating room personnel in a specialized, high‐acuity clinical setting and simultaneously examined an individual emotional resource and an organizational ethical resource. Additional strengths include the relatively large analytic sample, inclusion of personnel from five hospitals, established measurement instruments with good internal consistency in the present sample, and sensitivity analyses evaluating potential hospital‐level clustering.

Several limitations should be acknowledged. First, the cross‐sectional design precludes causal inference and does not establish temporal ordering. Second, all principal variables were collected using self‐report questionnaires at one time point, creating potential response, social‐desirability, and common method bias. Although Harman’s single‐factor test yielded a first‐factor variance of 31.42%, below the 50% criterion, this diagnostic cannot definitively exclude common method bias [22]. Third, convenience sampling within hospitals may have introduced selection bias, and the sample should not be considered statistically representative of all operating room personnel in Shiraz or other settings. Fourth, potentially relevant factors—including workload, burnout, autonomy, leadership style, staffing conditions, and organizational justice—were not measured and may confound or help explain the observed associations. Fifth, all participating hospitals were affiliated with one university. Although the ICC was low (0.024) and mixed‐model sensitivity analyses were materially consistent with OLS estimates, only five hospital clusters were available, limiting characterization of broader between‐hospital variation and generalizability.

Future research should prioritize multicenter studies across different healthcare systems, longitudinal designs, qualitative investigations of contextual influences on job involvement, and intervention studies evaluating whether changes in personal or organizational resources are followed by changes in job involvement. Mediation and moderation analyses may also clarify mechanisms involving leadership, organizational justice, autonomy, and workload.

### 4.3. Conclusion

Operating room personnel reported a generally favorable perceived ethical climate, while mean job involvement was slightly below the theoretical midpoint. Emotional intelligence and ethical climate were independently associated with greater job involvement, with ethical climate demonstrating the stronger association in the fully adjusted model. Viewed through JD‐R theory, emotional intelligence and ethical climate may represent distinct personal and organizational resources associated with employees’ psychological involvement in work. Their weak intercorrelation further suggests relatively independent pathways. Because the study was cross‐sectional, these relationships should not be interpreted causally. Longitudinal and intervention studies are needed to determine whether modifying emotional competencies, ethical workplace practices, or other job resources changes job involvement.

## Funding

This research received no specific grant from any funding agency in the public, commercial, or not‐for‐profit sectors.

## Conflicts of Interest

The authors declare no conflicts of interest.

## Data Availability

The data analyzed in this study are not publicly available because of confidentiality considerations. They may be obtained from the corresponding author upon reasonable request and subject to applicable institutional and ethical requirements.
